# Genotyping-by-Sequencing Reveals Low Genetic Diversity and Pronounced Geographic Structuring in the Endangered Medicinal Plant *Coptis chinensis* var. *brevisepala*

**DOI:** 10.3390/plants15030371

**Published:** 2026-01-25

**Authors:** Wenhao Zeng, Zihao Ye, Xi Liu, Haiping Lin, Jiasen Wu

**Affiliations:** 1 State Key Laboratory for Development and Utilization of Forest Food Resources, Zhejiang A&F University, Hangzhou 311300, China; zengwenhao@stu.zafu.edu.cn (W.Z.); lyxnmyyzh@163.com (Z.Y.); 2 Zhejiang Wuyanling National Nature Reserve Management Center, Wenzhou 325500, China; liuxiliushi@163.com

**Keywords:** *Coptis chinensis* var. *brevisepala*, genotyping-by-sequencing, genetic diversity, population structure, isolation by distance, isolation by environment

## Abstract

*Coptis chinensis* var. *brevisepala* W. T. Wang & P. G. Xiao is an endemic and endangered medicinal plant in China whose wild populations are rapidly declining under the combined pressures of overharvesting, climate change, and habitat fragmentation. Using genotyping-by-sequencing, we analyzed 87 individuals from 15 populations in Zhejiang Province, China, and identified 155,611 high-quality SNPs. The species exhibited low genetic diversity and strong genetic differentiation among populations with restricted gene flow (population-averaged *H*_o_ = 0.066, *H*_e_ = 0.067, *π* = 0.078, *F*_IS_ = 0.029, *F*_ST_ = 0.503, *N*m = 0.329, gRelMig = 0.136). Analysis of molecular variance showed that variation among populations accounted for 73.58% of the total genetic variation (*p* < 0.001). A phylogenetic tree, principal component analysis (PCA), and admixture analysis consistently resolved the 15 populations into two major groups, which could be further subdivided into four subgroups. Mantel and partial Mantel tests indicated that geographic isolation is the primary driver of genetic differentiation, while environmental factors such as ultraviolet radiation and low temperature may contribute to fine-scale divergence at local spatial scales. Furthermore, MMRR analysis provided further confirmation of the independent and dominant role of geographic isolation. This study provides key data on the genetic diversity and population structure of *C. chinensis* var. *brevisepala* and offers a genetic basis for developing regionally differentiated conservation strategies and promoting its sustainable utilization.

## 1. Introduction

Plant genetic diversity underpins species’ adaptive capacity and long-term evolutionary potential [1,2,3]. Under the combined pressures of climate change, habitat fragmentation, and intensifying anthropogenic disturbance, many wild plant populations are experiencing genetic erosion alongside demographic decline [2,4,5], a pattern that is particularly acute in high-value medicinal taxa [6,7]. Because genetic erosion can compromise population persistence and stress tolerance [1,3,8,9], quantifying genetic status and disentangling the relative roles of isolation by distance (IBD) and isolation by environment (IBE) have become central topics in conservation genetics and prerequisites for designing effective conservation and sustainable utilization strategies [2,10,11,12,13].

*Coptis chinensis* var. *brevisepala* W. T. Wang & P. G. Xiao is a perennial herb of the genus *Coptis* (Ranunculaceae) endemic to China and listed as a Class II nationally protected wild plant [14,15]. Its rhizomes are rich in isoquinoline alkaloids and have long been used in traditional Chinese medicine to manage inflammatory disorders [16,17,18,19]. However, due to its narrow distribution range, together with intensive harvesting driven by strong market demand and ongoing habitat destruction, wild populations have undergone rapid decline [20,21,22]. These processes increase the risks of genetic drift and inbreeding depression, thereby further accelerating the loss of genetic diversity [9,23,24,25,26]. Against this background, a systematic, genetics-based assessment of the patterns of genetic diversity and population structure of *C. chinensis* var. *brevisepala*, and the delineation of conservation units with differentiated management priorities, is of great importance for its long-term conservation and for future germplasm innovation [11,27].

To date, research on *C. chinensis* var. *brevisepala* has focused mainly on wild resource surveys and ecological niche modelling in parts of its range [15,22], phytochemical characterization and quality evaluation [18], DNA-based molecular identification [28], chloroplast genomes and phylogenetic relationships within *Coptis* [29,30,31], rhizosphere soil microbial communities [32], and techniques for ex situ introduction and cultivation [33]. At the genetic level, only preliminary studies based on chloroplast markers and simple sequence repeats (SSRs) have been conducted for a limited number of populations [20,34,35]. Such traditional markers generally suffer from limited locus numbers and low genome coverage, making it difficult to resolve the fine-scale genetic structure and demographic dynamics of species inhabiting complex mountain systems [36,37,38]. With the advent of high-throughput sequencing, genome-wide single-nucleotide polymorphisms (SNPs) can be generated at scale and provide dense, codominant markers for population genetic inference, and genotyping-by-sequencing (GBS) enables efficient SNP discovery and genotyping in non-model plants even without a reference genome [36,39,40,41,42,43,44,45,46]. Nevertheless, genome-wide SNP-based analyses of genetic diversity and structure in *C. chinensis* var. *brevisepala*, as well as quantitative evaluations of the relative roles of geographic isolation and environmental isolation in shaping its genetic differentiation, are still largely lacking.

In this context, the present study focuses on *C. chinensis* var. *brevisepala* populations from Zhejiang Province, China, which represents the core area of the species’ distribution. Using a high-quality SNP dataset generated by GBS in combination with multi-scale environmental variables, we aimed to conduct a comprehensive assessment of its genetic diversity and population structure. Because natural populations are small and fragmented, field sampling is inevitably constrained, underscoring the need for genome-wide markers while also motivating cautious interpretation of per-population diversity estimates. Given the species’ fragmented montane distribution, we hypothesized that geographic separation among mountain systems would be the primary driver of genetic differentiation. Specifically, we sought to (1) accurately evaluate the levels of genetic diversity in populations of *C. chinensis* var. *brevisepala* across Zhejiang Province; (2) elucidate the spatial patterns of genetic differentiation and population structure, and estimate the intensity of gene flow (*N*m) among populations; and (3) quantify the relative contributions of IBD and IBE to genetic differentiation, thereby identifying the key drivers of genetic structuring in this species. The findings will provide a robust scientific basis for designing regionally differentiated and hierarchical conservation schemes, as well as for promoting the sustainable utilization of this endangered medicinal plant.

## 2. Results

### 2.1. Sequencing Data and SNP Characteristics

A total of 87 individuals of *C. chinensis* var. *brevisepala* were genotyped using GBS, yielding 333.28 Gb of raw data, of which 264.13 Gb were retained after quality control. The mean Q30 score reached 96.30%, with an average GC content of 45.61% (41.62–53.29%), and clean reads accounted for 82.11% of the raw reads. These metrics indicate high overall sequencing quality, with adequate depth and consistency to support subsequent population genetic analyses.

Following standardized variant calling and standard filtering thresholds (minimum sequencing depth ≥ 2×, missing rate < 30%, and minor allele frequency, MAF ≥ 0.05), a total of 155,611 high-quality SNPs were identified. Among these loci, 116,009 were transitions (Ts) and 39,602 were transversions (Tv), giving a Ts/Tv ratio of 2.93. The Ts/Tv ratio is widely used as an internal diagnostic of SNP callset quality and supports the suitability of this dataset for downstream analyses of genetic diversity and population structure [47,48].

### 2.2. Levels of Genetic Diversity

Genetic diversity parameters were estimated for each of the 15 populations based on the high-quality SNP dataset (Table 1). Across populations, the mean expected heterozygosity (*H*_e_) was 0.067 (range: 0.024–0.124), while the mean observed heterozygosity (*H*_o_) was 0.066 (0.038–0.134), and the average nucleotide diversity (*π*) was 0.078 (0.029–0.143). The mean polymorphic information content (PIC) was 0.053 (0.019–0.099), and the mean Shannon’s diversity index (*I*) was 0.098 (0.034–0.184). All of these diversity indices reached their highest values in population QT1 and their lowest values in population TT, and *H*_o_ was slightly lower than *H*_e_ in just over half of the populations. The mean inbreeding coefficient (*F*_IS_) across populations was 0.029 (−0.016–0.077), with the highest *F*_IS_ recorded in population QY and the lowest in population TT.

### 2.3. Genetic Differentiation and Partitioning of Genetic Variation

**Figure 1:**
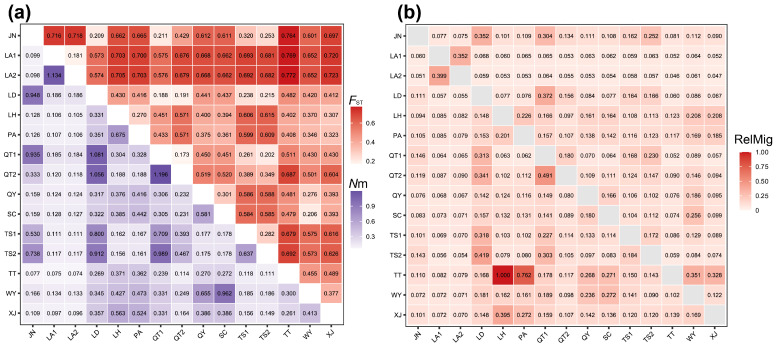
(**a**) Matrix heatmaps of the genetic differentiation coefficient (*F*_ST_) and gene flow (*N*m) among 15 populations of *C*. *chinensis* var. *brevisepala*. The upper triangle represents the inter-population genetic differentiation coefficient (*F*_ST_), and the lower triangle represents the inter-population gene flow (*N*m). (**b**) Heatmap of directional relative migration intensity (RelMig) among 15 populations of C. chinensis var. brevisepala inferred using divMigrate. Each cell shows the relative migration strength from the row population (source) to the column population (recipient). Values are normalized to 0–1, with 1 indicating the strongest directional connection in the dataset [49].

The mean pairwise *F*_ST_ among populations was 0.503, with the lowest genetic differentiation observed between QT1 and QT2 (*F*_ST_ = 0.173) and the highest between LA2 and TT (*F*_ST_ = 0.772; Figure 1). The corresponding estimate of gene flow (*N*m) averaged 0.329, with the greatest effective gene flow observed between QT1 and QT2 (*N*m = 1.196) and the weakest between LA2 and TT (*N*m = 0.074). Collectively, these results indicate that gene exchange among populations is generally limited, with appreciably stronger gene flow restricted to a few geographically proximate population pairs.

The relative migration analysis revealed (Figure 1b) that the mean relative migration rate among populations was 0.136, with the highest directional connectivity occurring from TT to LH (gRelMig = 1.000), whereas the reverse direction was lower (RelMig = 0.123). As shown in Figure 1a, LA2–TT had the highest *F*_ST_, and relative migration was low in both directions (LA2 to TT: RelMig = 0.046; TT to LA2: RelMig = 0.079), with LA2 to TT representing the lowest directional migration. The QT1–QT2 pair, which showed the highest Nm estimate in the preceding gene flow analysis (Figure 1a), showed directional asymmetry in relative migration (Figure 1b), with higher migration from QT2 to QT1 (RelMig = 0.491) than from QT1 to QT2 (RelMig = 0.180).

Analysis of molecular variance (AMOVA) (Table 2) revealed that genetic variation in *C. chinensis* var. *brevisepala* was predominantly partitioned among populations, with between-population divergence accounting for 73.58% of the total variance (Φ_ST_ = 0.7358, *p* = 0.001), whereas within-population variation among individuals contributed the remaining 26.42%. This high Φ_ST_ indicates pronounced population genetic structuring, corroborating the strong differentiation suggested by the pairwise *F*_ST_ and *N*m estimates.

### 2.4. Genetic Structure Analyses

#### 2.4.1. Phylogeny, PCA, and Admixture Analysis

**Figure 2:**
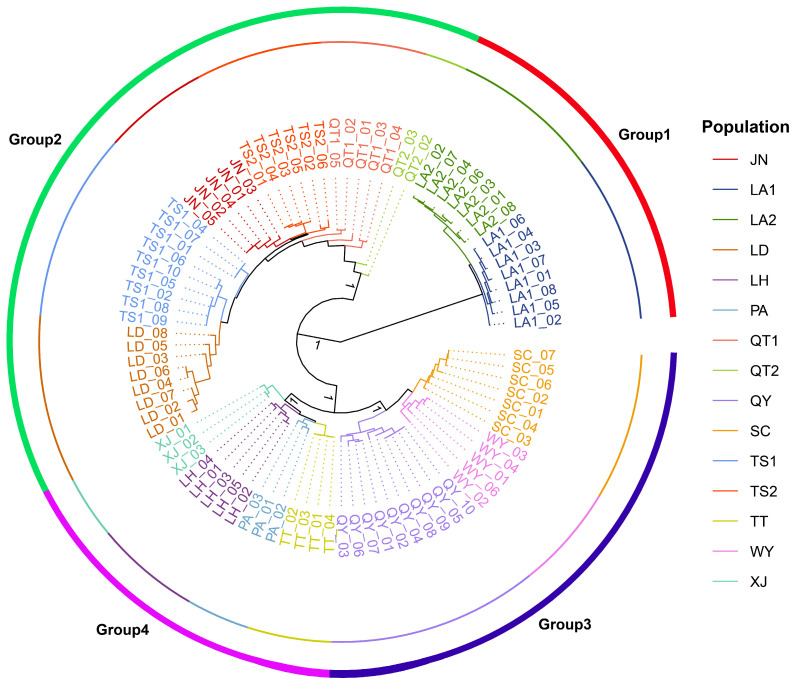
Phylogenetic tree of *C. chinensis* var. *brevisepala* based on SNP data from 87 individuals. Bootstrap support values are shown at major nodes and are expressed as proportions (0–1).

The phylogenetic tree (Figure 2) showed that individuals from the same population preferentially clustered together, and that all samples were clearly separated into two major genetic clades comprising four well-supported subclades. Clade I consisted solely of populations LA1 and LA2, which formed a tightly clustered sister group, whereas Clade II contained all remaining populations. Within Clade II, Subclade I comprised populations JN, QT1, QT2, TS1, TS2 and LD, while Subclade II included SC, WY, QY, LH, TT, PA and XJ. The latter was further resolved into two terminal subclades, with Terminal Subclade I comprising populations SC, WY and QY, and Terminal Subclade II comprising populations LH, TT, XJ and PA. These clustering patterns were highly consistent across the phylogenetic tree, PCA, and admixture analyses.

**Figure 3:**
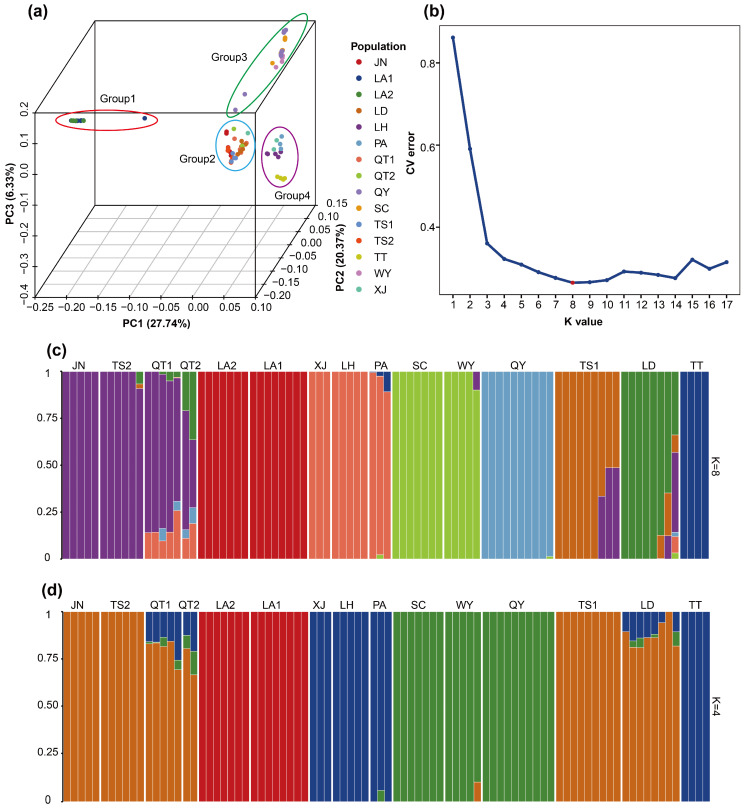
(**a**) Principal component analysis (PCA) of 87 samples of *C. chinensis* var. *brevisepala*; each point represents a single individual; (**b**) Cross-validation error for different K values in admixture analysis; (**c**) Stacked bar plots of individual ancestry coefficients at K = 8, with each color representing a distinct ancestral component; (**d**) Stacked bar plots of individual ancestry coefficients at K = 4, with each color representing a distinct ancestral component.

The principal component analysis (PCA) results (Figure 3a) were highly concordant with the phylogenetic tree (Figure 2). PC1, PC2 and PC3 explained 27.74%, 20.37% and 6.33% of the total genetic variation, respectively, together accounting for 54.44% (Figure 3a), and resolved four distinct genetic clusters. Along PC1, populations LA1 and LA2 formed a distinct cluster clearly separated from all remaining populations. Along PC2, the remaining samples were further partitioned into two clusters: one comprising populations TS1, TS2, LD, QT1, QT2 and JN, and the other including SC, WY, QY, TT, PA, LH and XJ. This latter cluster was further subdivided along PC3, with SC, WY and QY forming one group, and TT, PA, LH and XJ forming another.

Admixture analysis identified K = 8 as the optimal number of ancestral clusters based on cross-validation (CV error = 0.26; Figure 3b,c). However, this partitioning further subdivided several populations into multiple minor components, yielding a highly fragmented genetic structure with limited biological interpretability. By contrast, the assignment at K = 4 produced ancestral components that were most consistent with the known geographic distribution and with the clustering patterns inferred from the phylogenetic tree (Figure 2) and PCA (Figure 3a) and was therefore considered more biologically meaningful (Figure 3d). At K = 4, populations LA1 and LA2 were composed of the same ancestral component; TS1, TS2, JN, LD, QT1 and QT2 were dominated by a second ancestral component; SC, WY and QY were mainly characterized by a third component; and LH, XJ, PA and TT were primarily assigned to a fourth ancestral component.

Cluster-level AMOVA was further performed to statistically evaluate the four genetic clusters consistently inferred from the phylogenetic reconstruction, PCA, and Admixture analysis (K = 4) (Table 2b). The analysis showed that 65.37% of the total genetic variance was attributable to differences among clusters, whereas 34.63% was partitioned within clusters (Table 2b), yielding a high Φ_ST_ = 0.6537 (*p* = 0.001).

#### 2.4.2. Kinship and Linkage Disequilibrium Decay

**Figure 4:**
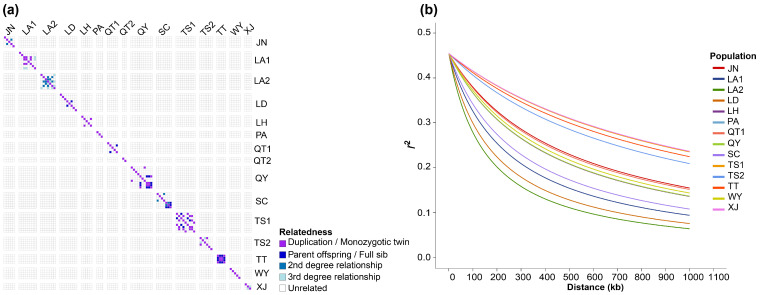
(**a**) Heatmap of pairwise kinship coefficients among 87 individuals from 15 populations of *C. chinensis* var. *brevisepala*; each cell represents an individual pair, and colour indicates the corresponding kinship class; (**b**) Genome-wide LD decay across 15 populations. The *x*-axis shows inter-SNP distance (kb; VCF-coordinate-based proxy distance), and the *y*-axis shows *r*^2^.

Kinship analysis (Figure 4a) showed that 98.72% of all pairwise comparisons were classified as unrelated (UN), and close-kin pairs were rare (Duplication/Monozygotic twin, 0.45%; Parent offspring/Full sib, 0.40%; second-degree relationships, 0.21%; third-degree relationships, 0.21%. After excluding self-comparisons, the overall mean kinship coefficient was approximately zero, with an average of 0.05 within populations and values close to zero among populations. Close-kin pairs occurred predominantly within populations TS1, TT, QY and LA1, and no clear signal of second-degree or higher relatedness was detected between populations.

Linkage disequilibrium (LD) decay analysis (Figure 4b) revealed marked differences in the rate of LD decay among populations in the rate at which *r*^2^ declined with increasing inter-SNP distance. Population LA2 exhibited the most rapid decay, with the shortest half-decay distance (158.92 kb), followed by populations LD and LA1. By contrast, PA, TS1 and XJ displayed the slowest LD decay, with r^2^ failing to decline to half of its initial value within 1000 kb, indicating the presence of extensive and persistent linkage disequilibrium in these populations. Notably, QT2 (*n* = 2) was excluded because no SNPs passed the LD filtering thresholds, precluding a reliable estimate.

### 2.5. Correlation Analysis of Genetic Distance with Environmental and Geographic Distances

#### 2.5.1. Relative Roles of Geographic and Environmental Isolation in Shaping Genetic Differentiation

**Figure 5:**
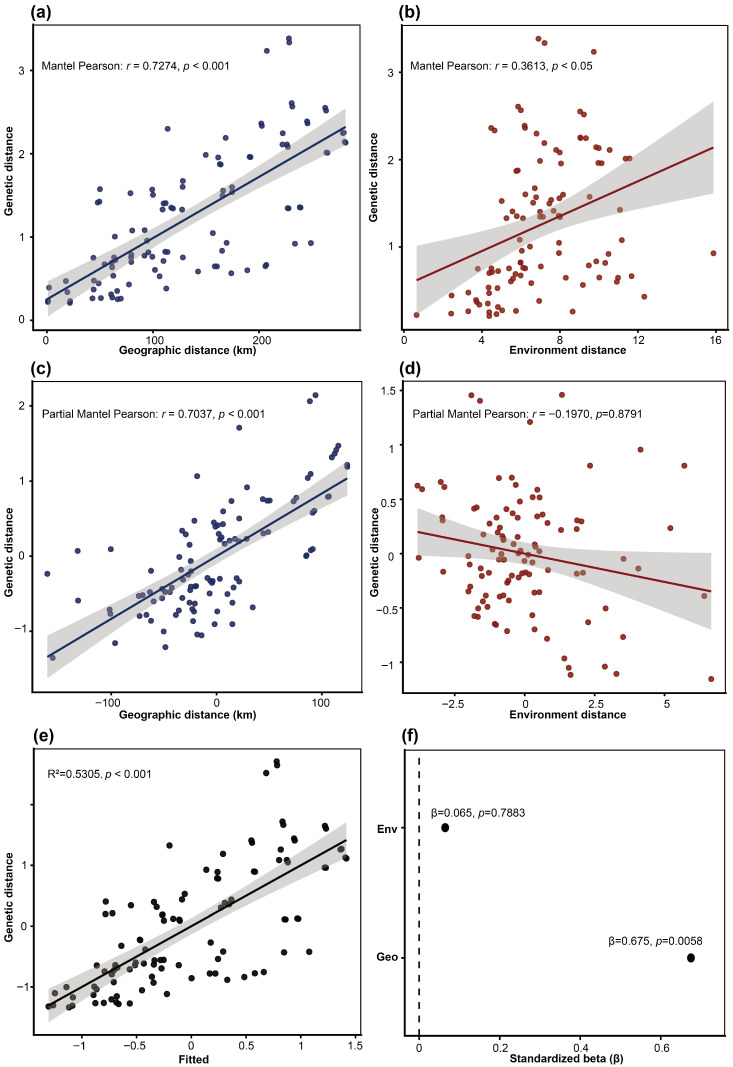
(**a**) Linear regression of genetic distance against geographic distance, with Mantel correlation statistics (isolation by distance, IBD); (**b**) Linear regression of genetic distance against composite environmental distance, with Mantel test statistics (isolation by environment, IBE); (**c**) Partial Mantel regression of genetic distance on geographic distance while controlling for environmental distance; (**d**) Partial Mantel regression of genetic distance on environmental distance while controlling for geographic distance; (**e**) Observed versus fitted values for the MMRR model, showing the relationship between the observed genetic distance and values predicted from geographic distance and EnvPC distance. Model fit statistics are shown in the panel (R^2^ and permutation *p*); (**f**) Standardized regression coefficients (β) from the MMRR model for geographic distance (Geo) and composite environmental distance in PCA space (Env). Points show standardized β, and labels report permutation-based *p*-values (9999 permutations).

Mantel tests (Figure 5a,b) revealed a highly significant positive correlation between genetic and geographic distance (*r* = 0.7274, *p* < 0.001), and a significant positive correlation between genetic and environmental distance (*r* = 0.3613, *p* < 0.05). In both cases, the regression lines exhibited positive slopes (Figure 5a,b), with the isolation-by-distance (IBD) relationship displaying a steeper slope and a more tightly clustered point cloud. Partial Mantel tests (Figure 5c,d) further showed that, after controlling for environmental distance, geographic distance remained strong and highly significant correlated with genetic distance (*r* = 0.7037, *p* < 0.001; Figure 5c), whereas the association between environmental distance and genetic distance, when controlling for geographic distance, was weak, negative and statistically non-significant (*r* = −0.1970, *p* = 0.8791; Figure 5d).

The multiple matrix regression with randomization (MMRR) showed a significant overall fit (R^2^ = 0.5305, *p* < 0.001; Figure 5e). Consistent with the partial Mantel results (Figure 5f), geographic distance exhibited a significant independent positive effect on genetic distance after accounting for composite environmental distance (β = 0.675, *p* = 0.0058). In contrast, the composite environmental PC distance showed no independent effect once geographic distance was controlled (β = 0.065, *p* = 0.7883).

#### 2.5.2. Correlation Between Geographic Distance and Genetic Distance

**Figure 6:**
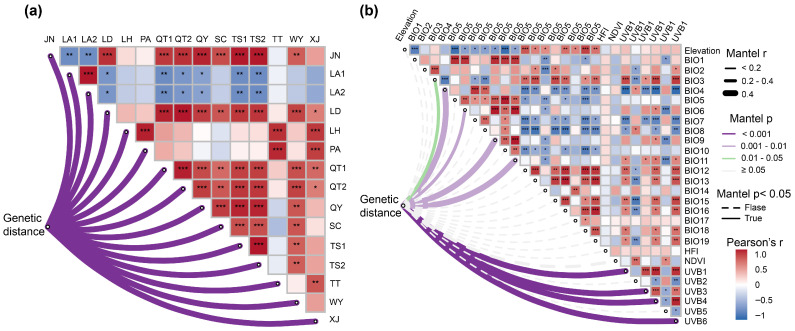
(**a**) Upper right panel: Heatmap of Pearson correlations between row vectors of the pairwise geographic distance matrix, quantifying the similarity of geographic distance profiles among the 15 populations of *C. chinensis* var. *brevisepala*. Lower left panel: leave-one-out Mantel test results showing the relationship between genetic distance and geographic distance when each population is omitted in turn; (**b**) Upper right panel: Pearson correlation heatmap among environmental variables. Lower left panel: single-factor Mantel test results for genetic distance versus environmental distance matrices. Asterisks indicate permutation-test significance levels: * *p* < 0.05; ** *p* < 0.01; *** *p* < 0.001.

Using a leave-one-population-out strategy, Mantel tests were iteratively performed (Figure 6a), and in all cases the correlation between genetic and geographic distance remained strongly positive and highly significant (*p* < 0.001). Pearson correlations between geographic distance profiles among populations (Figure 6a) showed that 60 pairwise comparisons exhibited significant correlation coefficients (*p* < 0.05), among which 34 displayed significant positive correlations (*p* < 0.01), indicating broadly similar geographic isolation patterns. For example, LA1–LA2 showed the highest correlation coefficients (*r* > 0.99). By contrast, 14 pairs, including LA1–TS2 and LA2–QY, showed significant negative correlations.

#### 2.5.3. Correlation Between Environmental Factors and Genetic Distance

Pearson’s correlation analysis among environmental variables (Figure 6b) showed that 166 out of 378 pairwise comparisons were significant (*p* < 0.05), indicating pronounced covariation among multiple environmental predictors. Among these, BIO9 and BIO11 exhibited the strongest correlation (*r* = 0.9938). Single-factor Mantel tests (Figure 6b) further revealed that nine environmental variables were significantly associated with genetic distance (*p* < 0.05). Among radiation-related variables, UVB1, UVB3, UVB4 and UVB6 all showed highly significant positive correlations (*p* < 0.001), with UVB3 displaying the strongest association (*r* = 0.6197). Five temperature-related bioclimatic variables (BIO11, BIO9, BIO6, BIO4 and BIO3) also exhibited significant positive correlations (*p* < 0.05), among which BIO11 had the highest correlation coefficient (*r* = 0.4663).

## 3. Discussion

### 3.1. Genetic Diversity and Implications for Population History

Based on the GBS-derived SNP dataset, populations of *C. chinensis* var. *brevisepala* exhibited a mean *H*_o_ of 0.066, a mean *H*_e_ of 0.067 and a mean *π* of 0.078 (Table 1). These values are substantially lower than those reported for most other endangered medicinal plants [44,50,51,52]. The analysis further revealed that the mean Ho was slightly lower than the mean He and that the average inbreeding coefficient *F*_IS_ was positive, a pattern that is consistent with the classic genetic signature of small effective population size, intensified genetic drift, and inbreeding-driven heterozygote deficiency [53,54]. Consistent with this, a previous SSR-based study also indicated a pronounced tendency toward selfing in *C. chinensis* var. *brevisepala* [20]. Like many medicinal plant species subjected to habitat loss and anthropogenic over-collection, such populations often have reduced effective population sizes [44,51,52]. Reduced Ne can intensify genetic drift and inbreeding, thereby generating heterozygote deficiency, consistent with theoretical expectations and empirical observations on the genetic consequences of small populations [55,56]. It is noteworthy that kinship analysis (Figure 4a) demonstrated an almost complete absence of second-degree or closer relationships among sampled individuals, and cross-population close-kin pairs were extremely rare, effectively ruling out substantial sampling of closely related individuals as a methodological artefact [57]. The reduced heterozygosity observed here therefore appears to reflect the intrinsic genetic status of natural populations rather than a sampling bias. Furthermore, Sample sizes were uneven among populations, and extremely small samples (e.g., QT2, *n* = 2) can downwardly bias within-population diversity estimates by failing to capture rare alleles. [58,59,60]. Accordingly, diversity metrics for such populations should be interpreted with appropriate caution and regarded as provisional [58,59,61]. LD decay patterns further corroborated this inference (Figure 4b). Populations LA1, LA2 and LD displayed relatively rapid LD decay, suggestive of higher historical recombination rates or comparatively larger effective population sizes, whereas populations PA, TS1 and XJ exhibited extensive and persistent linkage disequilibrium, a hallmark of long-term bottlenecks, inbreeding or severely restricted gene flow in small populations [23,53,62]. Consistent with the ecological traits of *C*. *chinensis* var. *brevisepala* [14,22], we observed during our preliminary sampling that this species has a low natural fruit set; it flowers in early spring, and the scarcity of pollinating insects results in insufficient pollination and consequent fruit abortion [63]. Under pollinator limitation, delayed autonomous selfing could provide reproductive assurance but may also elevate inbreeding if frequent [63]. These ecological hypotheses warrant explicit testing via pollinator surveys, pollen supplementation, and mating-system analyses. In addition, overharvesting and tourism development have further exacerbated habitat fragmentation [15]. Taken together with our genetic evidence, these findings suggest that this species is currently experiencing multiple, interacting pressures, including long-term demographic decline, genetic erosion and anthropogenic disturbance, underscoring the urgent need to implement zoned, hierarchical conservation and management measures.

### 3.2. Population Dynamics Revealed by Genetic Structure and Gene Flow

Multiple analyses consistently indicate a strong spatial structure in genetic variation in *C. chinensis* var. *brevisepala*. AMOVA revealed that 73.58% of the total genetic variation occurred among populations (Table 2a). The high Φ_ST_ value (Φ_ST_ = 0.7358, *p* = 0.001) indicates that most genetic variation is attributable to differences among populations. Φ_ST_ quantifies the proportion of total molecular variance explained by hierarchical subdivision [64]. Such a pattern is likely shaped by reduced connectivity among fragmented habitats and strong genetic drift in small, isolated populations [65]. The mean pairwise *F*_ST_ of approximately 0.503 reflects a high degree of genetic differentiation, while the corresponding estimate of gene flow (*N*m ≈ 0.329) is far below the theoretical threshold required to effectively counteract genetic drift [66,67,68,69]. It should be emphasized that *N*m inferred from *F*_ST_ under the equilibrium island model relies on assumptions of symmetric migration and migration–drift equilibrium, and thus serves as a heuristic indicator rather than a precise estimate of migration magnitude [67,68,69]. Relative migration analysis complements the symmetric Nm approximation by revealing that contemporary gene flow is both spatially heterogeneous and strongly directional among populations [49]. Overall migration was low (mean gRelMig = 0.136). Stronger migration signals were observed primarily between geographically adjacent population pairs (e.g., QT1–QT2), where migration was markedly asymmetric, whereas migration remained at a low level in more strongly differentiated pairs (gRelMig < 0.080) (Figure 1b). Notably, TT → LH represented the strongest standardized unidirectional edge in the network (gRelMig = 1.000), suggesting that movement may be concentrated along a limited number of dispersal pathways or reflect directional spread (Figure 1b). However, this signal may also be influenced by differences in effective population size, uneven sampling, and historical processes, and therefore warrants cautious interpretation. Taken together, these lines of evidence support the view that most populations of *C*. *chinensis* var. *brevisepala* have persisted under weak connectivity over the long term. Substantial gene exchange appears to occur only between geographically adjacent populations (e.g., QT1–QT2), whereas many other population pairs (e.g., LA2-TS1, separated by more than 200 km) are widely spaced. Because *C. chinensis* var. *brevisepala* flowers in early spring, when effective pollinators are scarce, long-distance pollen- and seed-mediated dispersal is less likely. Moreover, pronounced environmental heterogeneity and complex topography may further constrain gene flow, making it difficult to counteract the loss of genetic diversity caused by genetic drift and inbreeding [46,70].

The phylogenetic tree, PCA and admixture analysis consistently revealed a clearly geographically clustered genetic structure (Figure 2 and Figure 3). Populations LA1 and LA2 from the Tianmu Mountains in northwestern Zhejiang were clearly separated from the remaining populations from central and southern Zhejiang, forming two major groups that could be further resolved into four subgroups, within which individuals from the same mountain range or the same population preferentially clustered together. The complex mountainous terrain and large geographic distances evidently impede gene flow and reinforce regional isolation, a mechanism repeatedly identified as one of the primary drivers of spatial genetic structuring in plants [70,71,72,73,74]. In the admixture analysis (Figure 3b–d), the cross-validation procedure identified K = 8 as the statistically optimal model; however, at this value, several populations were further subdivided into minor components, yielding a highly fragmented structure with limited biological interpretability. By contrast, the clustering solution at K = 4 produced ancestry components that were most congruent with the geographic pattern, as well as with the phylogenetic tree and PCA results, and therefore was considered to have the strongest biological interpretability. Such a trade-off between statistical fit and biological interpretability is common in model-based clustering of population genetic data [75,76]. Most populations displayed highly homogeneous ancestry profiles, suggesting that they can be treated as largely independent management units. By contrast, populations QT1, QT2, TS1, TS2 and LD, which are geographically proximate, exhibited discernible admixture signals, implying weaker barriers to gene flow or historical secondary contact among them. To statistically validate this consistent four-cluster structure inferred from all three analyses, a cluster-level AMOVA was performed (Table 2b), attributing 65.37% of the variance to differences among clusters (ΦST = 0.6537, *p* = 0.001), providing statistical support for this partition as the major genetic subdivision in *Coptis chinensis* var. *brevisepala*. Such a pattern of “strong overall differentiation with locally restricted admixture” is a typical hallmark of species that have experienced long-term geographic isolation punctuated by infrequent, spatially localized gene exchange events [46,77].

### 3.3. Effects of Geographic Distance and Environmental Distance on Genetic Differentiation

Our Mantel analyses indicated that both geographic and environmental distance were significantly and positively correlated with genetic distance (Figure 5a,b), suggesting that geographic isolation and environmental heterogeneity each contribute to shaping the genetic structure of *C. chinensis* var. *brevisepala*. However, the steeper regression slope and more tightly clustered scatter of the IBD relationship (Figure 5a,b) imply a stronger explanatory power of geographic distance relative to environmental distance. Partial Mantel tests further reinforced this pattern (Figure 5c,d): after controlling for environmental distance, genetic distance remained highly significantly correlated with geographic distance, whereas, once geographic distance was held constant, the relationship between environmental and genetic distance became non-significant (Figure 5c,d). These results indicate that geographic isolation plays a predominant role in driving genetic differentiation, while the apparent effects of environmental isolation may be, to some extent, confounded by the spatial autocorrelation of environmental gradients [12,13,78,79]. It is important to note that, in montane landscapes, environmental gradients are often collinear with geographic distance and exhibit strong spatial autocorrelation [78,80]. Partial Mantel coefficients and associated significance tests can be influenced by shared spatial structure, potentially leading to biased inference; these results should therefore be interpreted with caution [81]. To further disentangle the independent contributions of geographic and environmental distances, we applied multiple matrix regression with randomization [82] (Figure 5e). The MMRR results were concordant with the partial Mantel tests: geographic distance exerted an independent positive effect on genetic distance, whereas the composite environmental PC distance showed no significant independent association with genetic distance once geographic distance was accounted for (Figure 5e). Taken together, these convergent lines of evidence indicate that geographic isolation is the predominant driver of genetic differentiation, while environmental effects may be intertwined with spatial structure or expressed primarily at finer spatial scales. The leave-one-out Mantel procedure (Figure 6a) showed that the IBD signal remained stable regardless of which population was removed, providing additional support that geographic distance is the primary driver of the observed genetic structure. High Pearson correlation values between row vectors of the pairwise geographic distance matrix (geographic distance profiles) were concentrated among pairs of populations that are geographically close or located within the same mountain range (Figure 6a). Given the limited seed dispersal capacity of *C. chinensis* var. *brevisepala*, neighboring populations are likely to share similar histories of dispersal and barrier effects, leading to relatively small genetic divergence, whereas populations separated by large spatial distances and complex mountainous barriers experience sharply reduced gene flow and accumulate substantial genetic differentiation over time—a pattern consistent with classical IBD dynamics [12,83].

Correlation analyses among environmental variables revealed that approximately 44% of pairwise comparisons were significant (Figure 6b), indicating pronounced collinearity among predictors. Although the IBE signal was not significant, single-variable Mantel tests still detected significant associations between genetic distance and several radiation-related variables (UVB1, UVB3, UVB4, UVB6), as well as temperature-related bioclimatic variables (BIO3, BIO4, BIO6, BIO9, BIO11), These univariate signals should be interpreted cautiously under strong collinearity and spatial autocorrelation, suggesting that environmental heterogeneity may exert a “fine-tuning” effect on genetic patterns at local scales [84]. Numerous studies have demonstrated that, in montane systems, ultraviolet radiation and low-temperature stress act as key selective agents driving spatial differentiation in genes associated with photoprotection, DNA repair and cold tolerance [13,74,84,85,86]. *C. chinensis* var. *brevisepala* is adapted to cool, shaded and highly humid understory habitats and appears to have limited environmental tolerance [22]. Taken together, our results suggest that geographic isolation is the dominant force underlying its genetic differentiation, while sensitivity to variation in light radiation and low-temperature regimes may contribute to population-specific local adaptation and fine-scale divergence.

### 3.4. Conservation and Management Implications

*C. chinensis* var. *brevisepala* exhibits a genetic pattern characterized by low diversity, high differentiation and weak gene flow. We therefore recommend that the distinct genetic groups identified in this study be treated as independent management units (MUs) [87,88]. In situ conservation, seed collection for population reinforcement and introduction–cultivation programmes should preferentially deploy germplasm within the same MU to reduce maladaptation and the practical risk of outbreeding depression that can arise when mixing highly divergent clusters [35,70]. For very small, low-diversity populations (e.g., TT in the Tiantai Mountains; LH and XJ in the Kuocang Mountains), priority actions include habitat restoration, demographic reinforcement, and immediate ex situ safeguarding (seed banking) of local germplasm. Notably, low diversity estimates may partly reflect limited sampling rather than the true genetic state of the populations and should therefore be interpreted cautiously. In practice, decisions on population reinforcement versus genetic rescue can follow a stepwise set of criteria: reinforcement should be used when compatible donor material exists within the same MU and under similar habitat conditions; genetic rescue should be considered only when inbreeding risk is high and local donors are unavailable, and should proceed stepwise via donor selection from the most genetically and environmentally similar sources, small-scale pilot translocations, and post-release fitness monitoring to detect any outbreeding-depression signals. Under ongoing climate warming, the establishment long-term monitoring plots will provide the evidence base for ex situ conservation and adaptive management [89,90].

## 4. Materials and Methods

### 4.1. Plant Materials

**Figure 7:**
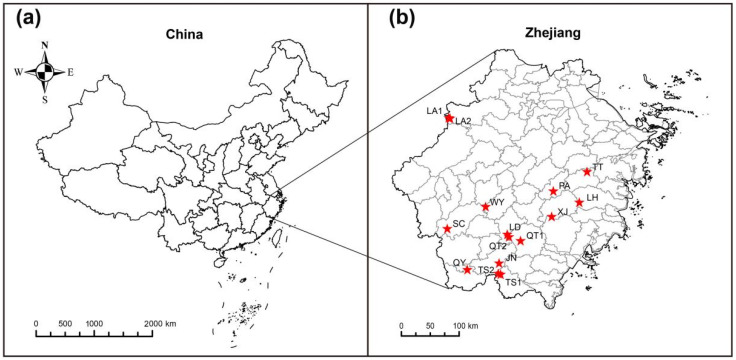
(**a**) Schematic map of the study area location; (**b**) Distribution of sampling sites for *C. chinensis* var. *brevisepala* (15 populations, 87 samples), where the red stars represent the sampling locations.

From February to April 2025, field sampling was conducted at 15 wild localities within the core distribution area of *C. chinensis* var. *brevisepala* in Zhejiang Province, China (Figure 7), yielding a total of 87 samples. For each sampling site, visually healthy plants free from obvious symptoms of disease or insect damage were selected, and fresh leaves were collected and immediately dried in silica gel for subsequent DNA extraction. Detailed sampling information is provided in Table 3.

### 4.2. Methods

#### 4.2.1. DNA Extraction, Library Construction, and Sequencing

Genomic DNA of *C. chinensis* var. *brevisepala* was extracted using a universal magnetic bead-based genomic DNA extraction kit [91]. DNA purity was assessed with a NanoDrop 2000 spectrophotometer (Thermo Fisher Scientific, Waltham, MA, USA), DNA concentration was quantified using a Quantus™ Fluorometer (Promega Corporation, Madison, WI, USA), and DNA integrity was evaluated by 1% agarose gel electrophoresis. Given the current lack of a reference genome for *C. chinensis* var. *brevisepala*, we adopted a reference-free (de novo) GBS strategy [43,45]. Briefly, 0.1–1.0 μg of genomic DNA per sample was digested with MseI. Barcoded P1/P2 adapters (6 bp barcode) compatible with Illumina (San Diego, CA, USA) sequencing were ligated to the restriction fragments. A second digestion with TaqaI was then performed to optimize tag representation. The resulting fragments were PCR-amplified and pooled across samples. Libraries were size selected by gel excision and purified using AMPure XP beads. Paired-end sequencing (PE150) was performed on an Illumina NovaSeq Xplus platform. All library preparation and sequencing were carried out by Shanghai Majorbio Bio-Pharm Technology Co., Ltd. (Shanghai, China).

#### 4.2.2. Sequencing Data Filtering and SNP Discovery

Raw sequencing reads were processed for quality control using Fastp v0.23.2 [92]. Adapter sequences were removed. Non-AGCT bases at the 5′ end were trimmed before quality trimming. Low-quality bases at read ends were trimmed using a Q20 threshold. Reads containing ≥10 ambiguous bases (N) were discarded. Reads shorter than 25 bp after trimming were removed. Clean reads were demultiplexed and assigned to individual samples using axeR (AXE) [93]. De novo locus assembly and variant discovery were performed using Stacks v2.66 [94]. Loci were assembled for each individual and then summarized by population using the populations module to generate consensus loci and estimate allele frequencies. SNPs were called jointly across all samples. For downstream analyses, we retained SNPs with a locus call rate > 70% (MISS < 30%) and a minimum locus depth > 2×, and minor allele frequency (MAF) ≥ 0.05 [94]. Hardy–Weinberg equilibrium (HWE) statistics were computed for quality assessment but were not applied as a hard filter, because population structure can generate genuine deviations from HWE [95].

#### 4.2.3. Population Genetic Analyses

All downstream analyses were performed using the filtered SNP dataset described above. We used the populations module in Stacks v2.66 [94] to estimate a suite of genetic diversity parameters, including observed heterozygosity (*H*_o_), expected heterozygosity (*H*_e_), polymorphism information content (PIC), Shannon’s information index (*I*), nucleotide diversity (*π*) and the fixation index (*F*_ST_). Gene flow (*N*m) was approximated from *F*_ST_ under the classical island model as *N*m = (1/*F*_ST_ − 1)/4 [68]. Directional relative migration was assessed in R v4.3.3 using divMigrate (diveRsity). Relative gene flow was expressed as standardized gRelMig values (0–1) [49]. Uncertainty was evaluated by bootstrap resampling (1000 replicates). Analysis of molecular variance (AMOVA) was performed in R v4.3.3 using the poppr package [96] following Excoffier et al. [64]. Significance was assessed by permutation testing (*n* = 999). A phylogenetic tree was reconstructed using the maximum-likelihood algorithm in FastTree 2 [97] under the GTR + Gamma substitution model (options-nt-gtr-gamma), with branch support estimated from 1000 bootstrap replicates. The resulting tree was subsequently visualized and annotated using the Interactive Tree of Life (iTOL) platform [98]. Genotypes in VCF format were converted using PLINK2 [99]. Principal component analysis (PCA) was performed with smartpca in EIGENSOFT [100] to summarize major axes of genetic variation. Pairwise kinship coefficients were estimated using the KING method implemented in PLINK2 [101]. This analysis identified close-kin individuals to avoid kinship bias and preserve data objectivity [57]. Population genetic structure and ancestry were inferred using ADMIXTURE v1.3.0 [75]. The optimal number of ancestral clusters (K) was identified by comparing K values from 1 to 17 based on the lowest cross-validation error. In this analysis, the random seed was fixed at 12,345. The final and biologically most plausible grouping scheme was established by integrating results from ADMIXTURE, phylogenetic tree, and PCA analyses. Linkage disequilibrium (LD) decay was characterized using PopLDdecay v3.42 [102]. For this analysis, the inter-SNP distance (kb) was calculated as ∣ΔPOS∣/1000 using variant coordinates from the internal scaffold (*sca1*) and was interpreted as a proxy distance scale under the reference-free pipeline.

#### 4.2.4. Geographic and Environmental Data Acquisition and Correlation Analysis

To investigate the relationships between environmental factors and patterns of genetic differentiation among populations, we quantified correlations among genetic, geographic and environmental distances. Bioclimatic variables (BIO1–BIO19) were obtained from the WorldClim database [103], UV-B radiation data were sourced from the glUV platform [104], the Human Footprint Index (HFI) was retrieved from Mu et al. [105], and normalized difference vegetation index (NDVI) data were obtained from the Resource and Environmental Science and Data Center of the Institute of Geographic Sciences and Natural Resources Research, Chinese Academy of Sciences. The environmental predictors used in this study are summarized in Table 4.

All environmental variables were standardized prior to analysis and used to construct a composite Euclidean environmental distance matrix. Genetic distance among populations was represented by linearized *F*_ST_ [*F*_ST_/(1 − *F*_ST_)] [106]. Pairwise geographic distances (km) between populations were calculated from their latitude and longitude coordinates using the Haversine great-circle formula, as implemented in the R package geosphere (R v4.3.3). Mantel and partial Mantel tests implemented in the vegan package were then used to evaluate isolation by distance (IBD; genetic vs. geographic distance) and isolation by environment (IBE; genetic vs. composite environmental distance), and to compare their relative contributions to genetic differentiation. Statistical significance was assessed using 9999 permutations. For IBD and IBE Mantel tests, 95% confidence intervals of Mantel’s r were obtained by bootstrap resampling of populations with replacement (*n* = 1000) and summarized as the 2.5th and 97.5th empirical quantiles. We further applied Multiple Matrix Regression with Randomization (MMRR) [82], modelling genetic distance as the response variable and geographic and composite environmental distances as explanatory variables. The overall model fit and individual regression coefficients (β) were assessed by permutation tests (9999 permutations) to mitigate non-independence among pairwise distance observations. To further dissect the influence of individual environmental factors, we constructed separate Euclidean distance matrices for each standardized environmental variable and performed single-factor Mantel tests between these matrices and the genetic distance matrix. The robustness of the IBD pattern was assessed using a leave-one-population-out strategy, in which Mantel tests were repeated iteratively after excluding each population in turn. All analyses were conducted in R v4.3.3, and scatterplots and correlation heatmaps were visualized using ggplot2 and related packages.

## 5. Conclusions

Based on GBS-derived SNP data, this study systematically reveals that *C. chinensis* var. *brevisepala* has low genetic diversity and exhibits a genetic pattern characterized by high differentiation and restricted gene flow. The genetic structure shows a pronounced pattern of geographic spatial clustering, with genetic variation mainly attributable to differences among populations; geographic isolation is the primary driver of genetic differentiation, whereas environmental factors such as ultraviolet radiation and low temperature act at secondary levels or local scales. We recommend treating the different genetic groups as independent management units. This study elucidates the genetic diversity and genetic structure of *C. chinensis* var. *brevisepala* and their influencing factors, and provides a genetic basis for implementing zoned, hierarchical conservation and the sustainable utilization of this species.

## Figures and Tables

**Table 1:** Genetic Diversity Levels of the Investigated Populations.

Population	*H*_o_	*H*_e_	*π*	*F*_IS_	PIC	*I*
JN	0.040	0.044	0.054	0.027	0.034	0.064
LA1	0.063	0.057	0.062	0.002	0.045	0.084
LA2	0.065	0.056	0.062	−0.002	0.044	0.083
LD	0.099	0.117	0.127	0.069	0.094	0.176
LH	0.049	0.054	0.063	0.028	0.043	0.079
PA	0.056	0.056	0.070	0.026	0.044	0.082
QT1	0.134	0.124	0.143	0.019	0.099	0.184
QT2	0.122	0.072	0.122	0.000	0.055	0.101
QY	0.046	0.071	0.076	0.077	0.056	0.105
SC	0.066	0.073	0.080	0.035	0.058	0.108
TS1	0.053	0.066	0.070	0.041	0.052	0.096
TS2	0.050	0.062	0.070	0.052	0.050	0.094
TT	0.038	0.024	0.029	−0.016	0.019	0.034
WY	0.061	0.080	0.091	0.070	0.064	0.118
XJ	0.048	0.043	0.057	0.014	0.033	0.061
Mean	0.066	0.067	0.078	0.029	0.053	0.098

*H*_o_: observed heterozygosity; *H*_e_: expected heterozygosity; PIC: polymorphism information content; *I*: Shannon’s information index; *π*: nucleotide diversity; *F*_IS_: fixation index.

**Table 2:** Hierarchical analysis of molecular variance (AMOVA) of *C. chinensis* var. *brevisepala*.

Source of Variation	Degrees of Freedom (DF)	Sum of Squares (SS)	Variance Components	Percentage of Variation (%)	*p*-Value	Φ_ST_
a. Population-based AMOVA (15 populations)						
Among Populations	14	2,312,873.09	27,098.89	73.58	0.001	0.7358
Within Populations	72	700,666.40	9731.48	26.42		
Total	86	3,013,539.5	36,830.36	100		
b. Cluster-based AMOVA (4 genetic clusters inferred from PCA, admixture analysis, phylogeny)						
Among clusters	3	1,783,572.53	27,967.30	65.37	0.001	0.6537
Within clusters	83	1,229,966.95	14,818.88	34.63		
Total	86	3,013,539.48	42,786.18	100		

**Table 3:** Sampling information for *C. chinensis* var. *brevisepala* populations in Zhejiang Province, China.

County	Township	Population	Elevation (m)	Number of Samples
Jingning	Dongkeng Town	JN	692	5
Lin’an	Qingliangfeng Town	LA1	1021	8
Lin’an	Qingliangfeng Town	LA2	895	7
Liandu	Zhangcun Township	LD	893	8
Linhai	Kuocang Town	LH	905	5
Pan’an	Dapan Town	PA	850	3
Qingtian	Jupu Township	QT1	780	5
Qingtian	Zhangcun Township	QT2	794	2
Qingyuan	Baishanzu Town	QY	1574	10
Suichang	Huangshayao Town	SC	800	7
Taishun	Siqian Town	TS1	1068	9
Taishun	Luoyang Town	TS2	724	6
Tiantai	Shiliang Town	TT	458	4
Wuyi	Liucheng Town	WY	1266	5
Xianju	Zhuxi Town	XJ	718	3

**Table 4:** Environmental variables used in this study.

Code	Description	Unit
BIO1	Annual Mean Temperature	°C
BIO2	Mean Diurnal Range	°C
BIO3	Isothermality	-
BIO4	Temperature Seasonality	-
BIO5	Max Temperature of Warmest Month	°C
BIO6	Min Temperature of Coldest Month	°C
BIO7	Temperature Annual Range (BIO5-BIO6)	°C
BIO8	Mean Temperature of Wettest Quarter	°C
BIO9	Mean Temperature of Driest Quarter	°C
BIO10	Mean Temperature of Warmest Quarter	°C
BIO11	Mean Temperature of Coldest Quarter	°C
BIO12	Annual Precipitation	mm
BIO13	Precipitation of Wettest Month	mm
BIO14	Precipitation of Driest Month	mm
BIO15	Precipitation Seasonality (Coefficient of Variation)	-
BIO16	Precipitation of Wettest Quarter	mm
BIO17	Precipitation of Driest Quarter	mm
BIO18	Precipitation of Warmest Quarter	mm
BIO19	Precipitation of Coldest Quarter	mm
UVB1	Annual Mean UV-B	J m^−2^·d^−1^
UVB2	UV-B Seasonality	J m^−2^·d^−1^
UVB3	Mean UV-B of Highest Month	J m^−2^·d^−1^
UVB4	Mean UV-B of Lowest Month	J m^−2^·d^−1^
UVB5	Sum of Monthly Mean UV-B during Highest Quarter	J m^−2^·d^−1^
UVB6	Sum of Monthly Mean UV-B during Lowest Quarter	J m^−2^·d^−1^
NDVI	Normalized Difference Vegetation Index	-
HFI	Human Footprint Index	-
Elevation		m

## Data Availability

The data presented in this study are available from the corresponding author upon reasonable request.
